# The effect of genital self-image on sexual satisfaction and stress in women after vaginal delivery

**DOI:** 10.1590/1806-9282.20240692

**Published:** 2024-10-07

**Authors:** Tuğçe Sönmez, Özlem Koç, Emine Alver

**Affiliations:** 1 Tarsus University, Faculty of Health Sciences, Department of Midwifery – Mersin, Turkey.; 2 Süleyman Demirel University, Faculty of Health Sciences, Department of Midwifery – Isparta, Turkey.

**Keywords:** Reproductive health, Sexuality, Women

## Abstract

**OBJECTIVE:**

Pregnancy, childbirth, and postpartum periods bring along biological, psychological, and social changes that could affect women's sexual health. The aim of this study was to evaluate the effect of genital self-image on sexual satisfaction and stress in women who had a vaginal delivery.

**METHODS:**

This descriptive and cross-sectional study was conducted online between June and September 2023 by using snowball sampling and the data collection forms prepared in the GoogleDocs program. Women who had a normal vaginal delivery were included, were within 6 weeks to 1 year postpartum, were able to use at least one of the social media networks (e-mail, WhatsApp, Facebook, and Instagram), and volunteered to participate in the research. Data were collected through the Personal Information Form, the "Female Genital Self-Image Scale," the "Golombok-Rust Inventory of Sexual Satisfaction," and the "Female Sexual Distress Scale-Revised."

**RESULTS:**

The average age of the participating women was 30.54±5.83 years (n=235). The participants’ mean scores were 18.94±2.92 for the Female Genital Self-Image Scale, 40.07±15.51 for the Golombok-Rust Inventory of Sexual Satisfaction, and 8.85±8.50 for the Female Sexual Distress Scale-Revised total. A statistically significant and negative relationship was found between Female Genital Self-Image Scale and Golombok-Rust Inventory of Sexual Satisfaction and Female Sexual Distress Scale-Revised total scores (r=-0.183, p<0.01; r=-0.387, p<0.01). Regression analysis (forward) was performed, and genital self-image was found to be the predictive factor affecting sexual satisfaction and stress.

**CONCLUSION:**

This study found that women had a medium level of genital self-image and sexual satisfaction and a low level of sexual distress.

## INTRODUCTION

Sexuality, an integral part of health, is affected by many factors throughout a woman's life. Particularly, pregnancy and childbirth processes bring along biological, psychological, and social changes that could affect women's sexual health^1,2^. Their appearance is perceived by women as an integral component of their sexuality^2^.

For many women, changes experienced during pregnancy, such as color changes in the genital area and increased vaginal discharge, and changes experienced with childbirth (episiotomy, scar, etc.), could cause dissatisfaction in the body or cause women to experience unwanted conditions in body image. Body image is a multifaceted and dynamic phenomenon that includes emotional (shame and dysphoria), cognitive (dissatisfaction and desire for change), and behavioral (avoidance and concealment) aspects of an individual's perceived physical response^3^. Dissatisfaction with body image is associated with negative genital self-image^4^. Genital self-image refers to individuals’ emotional and psychological perceptions of their appearance, health status, and genital organs^4,5^. Studies indicate that women have a more positive genital self-image if their partners’ perception of genital organs is good, while just the opposite case causes women to experience unsuccessful sexual intercourse, which could lead to sexual dysfunction in women^6^.

Women could experience increased body image anxiety, as well as negative effects on sexual functioning and quality of life, during vaginal delivery, especially in cases involving the use of surgical instruments, episiotomy, which is a common obstetric operation, or perineal trauma^3,7^.

Therefore, it is important for health professionals to know about the problems related to sexual functioning in the postpartum period, to plan preventive interventions for these problems, and to make appropriate approaches when necessary. This study aims to examine the effect of genital self-image on sexual satisfaction and sexual stress in women who had a vaginal delivery.

## METHODS

### Study design and participants

This study used a descriptive and cross-sectional design. It was conducted online between June and September 2023 in Turkey and utilized the snowball sampling method, one of the non-random sampling methods. Data collection forms were prepared using the GoogleDocs program. A total of 235 women who had a vaginal delivery were reached using the online questionnaire forms. Post-hoc analysis was performed after the study was conducted, and an effect size of 0.274 was obtained with 95% power and a 0.05 margin of error^8^. The power analysis (GPower 3.1 program) conducted showed that the collected data were sufficient^9^.

### Data collection

Data collection was performed using the Personal Information Form, the "Female Genital Self-Image Scale (FGSIS)," the "Golombok-Rust Inventory of Sexual Satisfaction (GRISS)," and the "Female Sexual Distress Scale (FSDS-R)."

### Personal information form

The Personal Information Form prepared by the researchers consisted of 17 questions^1,3,4,6^. The form collects data about age, education, spouse's age and education, duration of the marriage, income level; number of pregnancies, births, stillbirths, and curettages; presence of episiotomy during childbirth; and satisfaction with the body, frequency of sexual intercourse, and the use of contraception in the postpartum period.

### Female Genital Self-Image Scale

The FGSIS was developed by Herbenick and Reece^10^ to measure women's genital self-perception. The Turkish validity and reliability of the scale were conducted by Karadeniz^11^. The scale consists of seven items responded on a 4-point Likert scale. The scores to be obtained from the scale range between 7 and 28, with a higher total scale score indicating a positive genital self-image. Cronbach's alpha value was found to be 0.88 in the original validity and reliability study of the scale^10^. In this study, Cronbach's alpha value was found to be 0.70.

### Golombok-Rust Inventory of Sexual Satisfaction-female form

The GRISS was developed by Rust and Golombock^12^. A Turkish standardization, validity, and reliability study was conducted by Tuğrul et al.^13^. The scale consists of 28 items and 7 sub-scales and includes two forms prepared for men and women. The sub-scales of the female form include frequency, communication, satisfaction, avoidance, touch, vaginismus, and anorgasmia. Higher scores indicate deterioration in sexual functioning and the quality of the relationship. Cronbach's alpha value was found to be 0.87 in the validity and reliability study of the scale for the female form^12^. Cronbach's alpha value for this study was found to be 0.88.

### Female Sexual Distress Scale-Revised

The scale was developed by Derogatis et al. in 2002 to evaluate women who had sexual dysfunction^14^. Kitiş et al.^15^ revised the Female Sexual Distress Scale in 2019. When they respond to the scale, women are asked to select the number that describes the frequency of discomfort from sexual problems experienced within the last 30 days. Cronbach's alpha coefficient of the revised form ranges between 0.87 and 0.93^14,15^. In this study, Cronbach's alpha coefficient was found to be 0.90.

### Statistical analysis

The data were analyzed in the IBM SPSS Statistics 25.0 program. Frequency analysis was performed to determine socio-demographic and obstetric characteristics. Independent sample t-tests and ANOVA were performed to determine the scale distributions according to socio-demographic and obstetric characteristics. Min–max, mean, and standard deviation analyses were performed for scale scores. Pearson correlation and linear regression analyses were performed between scale total scores.

### Ethics statement

This study was approved (number: 2023/19; date: 25/05/2023) by the Non-invasive Clinical Studies Ethics Committee at Tarsus University.

## RESULTS

Table 1 demonstrates the distribution of women according to demographic and obstetric characteristics. The average age of the participating women was 30.54±5.83 years. The results showed that 77.0% had an episiotomy/incision/stitch during vaginal delivery, 62.6% were satisfied with their body in the postpartum period, and 42.6% had sexual intercourse 2–3 times a week. A significant relationship was found between the current frequency of sexual intercourse and FGSI, GRISS, and FSDS-R (F=4.707, p=0.000; F=5.979, p=0.000; F=5.866, p=0.000) (Table 1).

**Table 1:** Distribution of scale scores according to descriptive and obstetric characteristics (n=235).

		n (%)	FGSI	GRISS	FSDS-R
Undergoing episiotomy/incision/stitch during vaginal delivery					
	Yes	181 (77.0)	18.76±2.84	39.82±15.55	9.04±8.83
	No	54 (23.0)	19.53±3.13	40.90±15.50	8.18±7.32
Test (p)			t=-1.702, p=0.090	t=-0.452, p=0.653	t=0.655, p=0.513
Being satisfied with the body					
	Yes	147 (62.6)	19.87±2.63	39.20±15.95	7.17±6.99
	No	88 (37.4)	17.39±2.73	41.54±14.72	11.65±9.98
	Test (p)		t=6.858, p=0.001	t=-1.114, p=0.267	t=-4.042, p=0.001
Frequency of sexual intercourse					
	Once a week	77 (32.8)	18.00±2.80	42.90±12.79	10.94±8.85
	Twice a week	28 (11.9)	18.89±2.76	46.42±14.64	9.39±7.53
	Two to three times a week	100 (42.6)	19.65±2.72	34.47±15.09	6.19±6.29
	More than three times a week	3 (1.3)	23.33±3.51	60.00±14.00	2.33±3.21
	Once a month	15 (6.4)	18.13±2.85	44.50±16.49	15.86±13.00
	No active sexual life	12 (5.1)	19.16±3.58	43.58±21.05	9.16±10.42
Test (p)			F=4.707, p=0.000	F=5.979, p=0.000	F=5.866, p=0.000
		Mean±SD			
Age		30.54±5.83			
Pregnancies		2.36±1.39			
Births		1.98±1.12			
Stillbirths		0.17±0.95			
Curettages		0.31±0.70			

Bold: p<0.001. FGSI: female genital self-image; GRISS: Golombok-Rust Inventory of Sexual Satisfaction; FSDS-R: Female Sexual Distress Scale; SD: standard deviation.

A statistically significant and negative relationship was found between FGSI and GRISS and FSDS-R total scores (r=-0.183, p<0.01; r=-0.387, p<0.01) (Table 2).

**Table 2:** Scale correlations.

SCALES***		1	2	3
(1) FGSI	r	1	-0.183**	-0.387**
	p	–	0.005	0.000
(2) GRISS	r	-0.183**	1	0.370**
	p	0.005	-	0.000
(3) FSDS-R	r	-0.387**	0.370**	1
	p	0.000	0.000	–

** p<0.01,

*** Pearson correlation analyses;

r: correlation coefficient (r=0.00–0.25 very weak; r=0.26–0.49 weak; r=0.50–0.69 medium; r=0.70–0.89 high; r=0.90–1.00 very high). FGSI: female genital self-image; GRISS: Golombok-Rust Inventory of Sexual Satisfaction; FSDS-R: Female Sexual Distress Scale.

Regression analysis (forward) was performed to determine the effect of women's genital self-image on sexual satisfaction and sexual stress. This regression model explains the effect of genital self-image on women's sexual satisfaction by 13% (R^2^=0.139, p<0.001). Genital self-image was determined to be a predictive factor for sexual stress. This regression model explained the effect of genital self-image on women's sexual stress by 23% (R^2^=0.239, p<0.001) (Table 3).

**Table 3:** Effect of women's genital self-image on sexual satisfaction and sexual stress (n=235).

								95%CI	
GRISS	B	SE	β	t	p	R	R^2^	Lower	Upper
Constant	39.290	7.180		5.472	<0.001	0.37	0.13	25.144	53.437
FGSI	-0.258	0.350	-0.049	-0.735	0.463	3	9	-0.948	0.433
FSDS-R	B	SE	β	t	p	R	R^2^	Lower	Upper
Constant	19.825	3.681		5.386	<0.001	0.48	0.23	12.572	27.077
FGSI	-0.940	0.169	-0.326	-5.579	<0.001	9	9	-1.272	-0.608
							Linear regression		

Bold: p<0.001. CI: confidence interval; GRISS: Golombok-Rust Inventory of Sexual Satisfaction; FGSI: female genital self-image; FSDS-R: Female Sexual Distress Scale.

## DISCUSSION

This study found a significant positive relationship between those who were satisfied with their postpartum bodies and genital self-image and a significant negative relationship in terms of sexual stress. A significant relationship was detected between the women's current sexual frequency and genital self-image, sexual satisfaction, and sexual stress.

Research suggests that women's genital dissatisfaction (e.g., the appearance of the vulva) is associated with lower sexual esteem, lower sexual satisfaction, lower sexual functioning, more pain during intercourse, and higher sexual stress^16,17^. Amos and McCabe^18^ reported that women with a positive genital self-image felt more sexually attractive, had sexual intercourse more frequently, and were more satisfied with their sexual relationships.

In their study that involved women in the early postpartum period, Bektaş and Öcalan^4^ found that the majority of them had a positive genital self-image, and women who liked their body as a whole in the postpartum period had a positive perception of genital self-image. Similarly, Komarnicky et al.^19^ reported a significant, positive relationship between genital self-image and body image in women. Jawed-Wessel et al.^8^ found that women who had a vaginal delivery had a lower genital self-image than women who had a cesarean section. Besides, as cesarean section decreases some traumas in the genital region, it is reported to enable a protective effect on problems caused by sexual activity^9^. Some changes in the woman's body image in the postpartum period, practices performed during delivery (episiotomy, laceration, etc.), breastfeeding, sexual behavior, and body weight changes affect satisfaction with the body.

This study found that women had moderate-level positive genital self-image and problems in the satisfaction, vaginismus, and anorgasmia sub-scales of the sexual satisfaction scale. Participating women were found to have a moderate level of sexual stress. In their study on factors influencing stress, anxiety, and depression among Iranian pregnant women: the role of sexual distress and genital self-image, Keramat et al.^19^ found that the women had a moderate level of genital self-image and a low level of sexual stress. Eftekhar et al.^6^ reported that the genital self-image scale mean score was lower in the experimental group compared to the control group. Vosoughi et al.^20^ found that the mean scores of genital self-image and sexual stress were similar in the experimental and control groups. The women who participated in the study conducted by Bay and Akın^21^ were found to have a moderately positive genital self-image. A comparison of the studies revealed similar results.

This study found a significant negative relationship between women's genital self-image, sexual satisfaction, and sexual stress. Besides, there was a significant positive relationship between sexual satisfaction and sexual stress. Berman et al.^22^ investigated the relationship between genital self-image and sexual functioning and found that positive genital self-image was negatively associated with sexual dysfunction.

The qualitative study conducted by Olsson et al.^23^ found that women's sexual desire decreased, and they experienced sexual stress because they perceived their vagina to be loose after childbirth. In another qualitative study, women interpreted their genital self-image in the context of their relationships with their partners and their healthy sexual lives. Women reported that good or bad genital self-image positively or negatively affected their relationships with their partners as well as their sexual satisfaction. Another study found that sexual satisfaction increased with an increase in the positivity of genital self-image^21^.

This study found that genital self-image was a predictive factor for sexual satisfaction and sexual stress and explained the effect of genital self-image on women's sexual satisfaction and sexual stress by 13 and 23%, respectively. Fudge and Byers^24^ reported that sexual comfort, body image, and positive genital image were the factors explaining approximately 35.0% of genital self-image (FGSI). Bektaş and Öcalan^4^ reported the factors explaining 19.5% of female genital self-image as age, self-esteem, and body image. These variables are accepted as important predictors of female genital self-image. Studies show that women's genital self-image is a determinant of sexual functions such as sexual satisfaction and sexual experience^21,25^. When the research findings and literature results are considered, a positive genital self-image seems to increase positive sexual functioning and satisfaction levels and reduce sexual distress.

### Limitations of the study

The limitations of this study are that it is not a comparative study and that it includes only women who had a vaginal delivery. The strengths of the study are that the literature includes no similar studies, and the effect of female genital self-image on sexual satisfaction and distress was evaluated using correlation and regression.

## CONCLUSION

This study found that women had a medium level of genital self-image and sexual satisfaction and a low level of sexual distress. In line with these results, it is important for all women to have a positive childbirth experience and take the necessary measures to prevent trauma. Genital self-image can be improved by preventing unnecessary surgical operations during labor and the postpartum period.
